# The Influence of Deposition Methods of Support Layer on Cordierite Substrate on the Characteristics of a MnO_2_–NiO–Co_3_O_4_/Ce_0.2_Zr_0.8_O_2_/Cordierite Three Way Catalyst

**DOI:** 10.3390/ma7096237

**Published:** 2014-09-02

**Authors:** Phuong Thi Mai Pham, Minh Thang Le, Tien The Nguyen, Els Bruneel, Isabel Van Driessche

**Affiliations:** 1 Laboratory of Environmental Friendly Materials and Technology, Advanced Institute of Science and Technology, Hanoi University of Science and Technology, 1 Dai Co Viet, Hanoi 10000, Vietnam; E-Mails: maiphuong1985@gmail.com (P.T.M.P.); thetienhdbk@gmail.com (T.T.N.); 2 Department of Organic and Petrochemical Technology, School of Chemical Engineering, Hanoi University of Science and Technology, 1 Dai Co Viet, Hanoi 10000, Vietnam; 3 Department of Inorganic and Physical Chemistry, SCRiPTS, Ghent University, Krijgslaan 281-S3, Gent 9000, Belgium; E-Mail: els.bruneel@ugent.be

**Keywords:** cordierite, oxidation, propylene, NO, CO, manganese, nickel, cobalt oxide catalysts, ceria—zirconia

## Abstract

This paper compares different coating methods (*in situ* solid combustion, hybrid deposition, secondary growth on seed, suspension, double deposition of wet impregnation and suspension) to deposit Ce_0.2_Zr_0.8_O_2_ mixed oxides on cordierite substrates, for use as a three way catalyst. Among them, the double deposition was proven to be the most efficient one. The coated sample shows a BET (Brunauer–Emmett–Teller) surface area of 25 m^2^/g, combined with a dense and crack free surface. The catalyst with a layer of MnO_2_–NiO–Co_3_O_4_ mixed oxides on top of the Ce_0.2_Zr_0.8_O_2_/cordierite substrate prepared by this method exhibits good activity for the treatment of CO, NO and C_3_H_6_ in exhaust gases (CO conversion of 100% at 250 °C, C_3_H_6_ conversion of 100% at 400 °C and NO conversion of 40% at 400 °C).

## 1. Introduction

Nowadays, environmental pollution attracts a lot of attention and, especially, the exhaust of vehicles poses significant abatement problems. In response to the challenges associated with treatment of this exhaust, three-ways catalysts (TWC) have been widely used to reduce pollutant emission from gasoline engine powered vehicles [1]. The TWC is generally composed of a ceramic substrate, a support layer, and an active layer of noble or transition metals. Many methods can be used to deposit these layers, depending on the properties of the substrate and the nature of the layers.

As a substrate material, cordierite exhibits excellent properties (high mechanical and thermal stability, low pressure drop, low thermal expansion coefficient), its most important disadvantage is the impermeability for gases and fluids [1].

For years, popular support materials such as zeolites and gamma-alumina, all possessing a high surface area, were deposited on top of the cordierite substrate by *in situ* synthesis techniques [2,3], such as suspension, slurry dip-coating [4,5,6] or sol-gel methods [7].

*In situ* combustion synthesis has already reported to be an efficient, quick, cheap and straightforward preparation process, suitable for producing a layer of catalyst with excellent adherence on the ceramic substrate [2,3]. For this synthesis, the catalytic loading content (up to 10%) and thickness of the catalyst layer are dependent on the concentration of the mother solution. In general, the surface areas of the catalysts remain small although an improvement compared to the original ceramic is observed [8].

The slurry method has also been applied to coat different powders such as γ-Al_2_O_3_, ZrO_2_, TiO_2_, CeO_2_ on cordierite ceramics. It was shown that a homogeneous and good adhesive coating layer on the ceramic substrate could be obtained providing that the slurry particle sizes are small (<2 μm) [9]. γ-Al_2_O_3_ was also deposited on cordierite from sols of colloidal boehmite [7,10] or γ-Al_2_O_3_ itself [10], by a sol-gel method. The viscosity of the solution, which was adjusted by binders and sol concentration, influenced significantly the loading content and thickness of the layer (5 μm) [7]. Compared with boehmite precursors, the wash coating of cordierite monoliths with γ-Al_2_O_3_ suspensions of suitable particle size allows a higher alumina loading, and the deposition of a homogeneous wash coat layer with good adhesion properties and surface areas of about 50 m^2^/g [10]. When the coating layer is zeolite (ZSM-5), the *in situ* crystallization method was chosen, in which zeolite can be deposited by direct crystallization from a gel on substrates placed together with the gel in an autoclave. The thickness of the layer varied from 70–100 μm. For the samples with a dense layer of zeolite, the adhesion was good but in some cases, less dense films were formed, which showed delamination [2]. Some modifications of the *in situ* crystallization method have been reported [3], where a seeding step was performed prior to the *in situ* crystallization of ZSM-5 by hydrothermal synthesis. According to this report, after the first synthesis, the cordierite substrate gained 28 wt%, and 11 wt% more after the second. The porosity of the final coated cordierite was 16%.

If a CeO_2_-based material is chosen as a support layer, this can act as a buffer against the considerable oscillations in the air-to-fuel ratio, owing to their oxygen storage and release function. Previous research has shown that the availability of CeO_2_ can improve catalytic properties [11,12]. Among all ceria-related materials, ceria—zirconia mixed oxides have attracted increasing interest due to their good thermal resistance to sintering and superior oxygen storage capacity [1]. Although many literature studies have investigated the role of ceria—zirconia mixed oxides as a support for the active phase [13,14,15,16], only limited information about coating procedures for this mixed oxide on the substrate is available. This coating step however may be a key issue for catalytic applications, determining the catalyst performance and life time.

A systematic comparison between the above mentioned methods in the preparation of three way catalysts is lacking. Some important properties of these catalysts such as their surface area haven’t been fully studied. Moreover, only few publications presented the catalytic activity of the whole catalyst complexes including ceramic substrate, support and catalytic active phase, as well as the influence of the impregnation method on the catalytic activity of the catalyst complex [3,10,17]. These publications only focus on several single treatments of toxic gases such as the treatment of NO [3], the treatment of isooctane using a Ni/γ-Al_2_O_3_/cordierite catalyst [10], or the absorption of SO_2_ [17].

Therefore, in this paper, we study and compare different deposition methods (*in situ* solid combustion, hybrid deposition, secondary growth on seed, suspension double depositions of wet impregnation and suspension) for coating Ce_0.2_Zr_0.8_O_2_ mixed oxide on cordierite tablets with the aim of finding the best method to produce stable MnO_2_–NiO–Co_3_O_4_/Ce_0.2_Zr_0.8_O_2_/cordierite catalyst complexes, exhibiting good surface area and high catalytic activity for the simultaneous treatment of all hydrocarbons, CO and NO in the exhaust gases. Ce_0.2_Zr_0.8_O_2_ mixed oxide was chosen as it was known that the remaining stable structure and high oxygen storage capacity at high temperatures (even at 1000 °C) [18,19,20], will allow the application of the catalytic converter in an engine at all working conditions (even when a thermal shock occurs). A MnO_2_–NiO–Co_3_O_4_ (mole ratio 2:3:3) catalytic active phase was chosen since it was found from our previous unpublished works that this catalyst exhibited a superior activity for the simultaneous treatment of hydrocarbon, CO and NO compared to other investigated mixed oxide catalysts.

## 2. Experimental Section

### 2.1. Materials

#### 2.1.1. Preparation of Cordierite Pellets

Cordierite was prepared from suitable compositions of kaolin, MgO and Al_2_O_3_. Kaolin was obtained from Yen Bai province (Vietnam), which was activated in a hydrochloric solution 36 wt% for 3 h prior to use. For each 20 g of activated kaolin, 2.542 g MgO and 1.0914 g Al_2_O_3_ was added. Afterwards, the components were ground, mixed, and dried before making pellets using a Carver hydraulic press with a pressed force of 10^5^ N for 5 min. The pellets were calcined at 1250 °C for 3 h. Because of its extremely low surface area, this cordierite must be pre-treated in the acid solution at severe conditions. The cordierite pellets, which have a diameter of 5 mm and a thickness of 2 mm, were immersed in 100 mL solution of HCl 36 wt% at 90 °C for 8 h. The acid solution was replaced with fresh solution every hour for extended treatment.

The treated pellets were washed thoroughly with distilled water until a neutral pH was reached. Afterwards, they were dried at room temperature. The obtained samples were assigned as CordxH8 (cordierite treated by HCl for 8 h).

#### 2.1.2. Preparation of Ce_0.2_Zr_0.8_O_2_ Powder

For the preparation of the Ce_0.2_Zr_0.8_O_2_ powder, we used a hydrothermal method. 1.6 mmol Ce(NO_3_)_3_·6H_2_O (98.5%, Merck, Darmstadt, Germany) and 6.4 mmol ZrOCl_2_·8H_2_O (99.0%, Merck) were dissolved with 16 mmol urea—CH_4_N_2_O (98%, Merck) in 80 mL H_2_O. The solution was then stirred until complete solubility. The obtained solution was poured into an autoclave, which was then maintained at 160 °C for 24 h. The obtained light-yellow precipitate was washed with distilled water until constant pH and dried at 80 °C. The BET (Brunauer–Emmett–Teller) surface area of this as-prepared Ce_0.2_Zr_0.8_O_2_ powder was about 200 m^2^/g.

### 2.2. Deposition Methods of Ce_0.2_Zr_0.8_O_2_ Support on Cordierite Substrate

**Figure 1:**
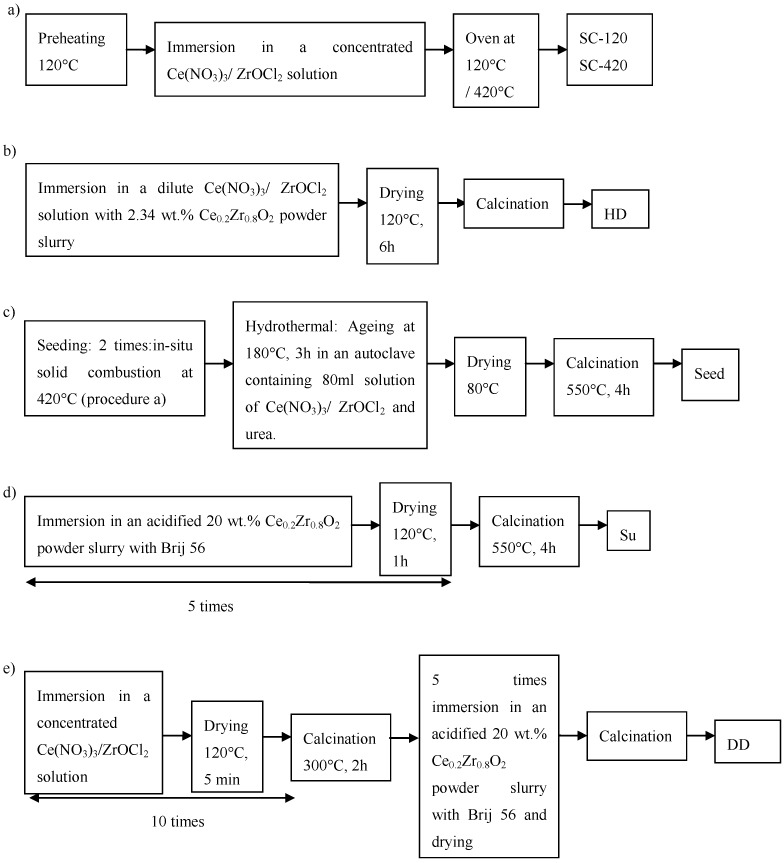
Deposition methods of Ce_0.2_Zr_0.8_O_2_ support on cordierite substrate: (**a**) *In situ* solid combustion; (**b**) Hybrid deposition; (**c**) Secondary growth on Ce_0.2_Zr_0.8_O_2_ seeds; (**d**) Suspension; (**e**) Double deposition combining wet impregnation and suspension.

A Ce_0.2_Zr_0.8_O_2_ support layer was deposited on the cordierite substrate by different methods, summarized in Figure 1.

#### 2.2.1. *In Situ* Solid Combustion (SC-120 and SC-420)

Cordierite pellets were preheated at 120 °C or 420 °C, immersed in an aqueous solution of 0.3 M Ce(NO_3_)_3_ and 1.2 M ZrOCl_2_ and dried at respectively 120 °C or 420 °C. These samples, further symbolized as, respectively, SC-120 and SC-420, are then air blown to remove loose powder.

#### 2.2.2. Hybrid Deposition (HD)

Cordierite pellets were dipcoated in an aqueous slurry of 2.34 wt% of Ce_0.2_Zr_0.8_O_2_ powder, 10^−5^ M Ce(NO_3_)_3_, 4 × 10^−5^ M ZrOCl_2_ and 2.8 M HNO_3_, dried and calcined at 550 °C for 3 h.

#### 2.2.3. Secondary Growth on Ce_0.2_Zr_0.8_O_2_ Seeds (Seed)

Seeds were grown by completing the solid combustion process at 420 °C twice. In the hydrothermal synthesis step, the seeded substrate was autoclaved in 80 mL of a 0.02 M Ce(NO_3_)_3_, 0.08 M ZrOCl_2_ and 0.2 M urea solution. After ageing at 180 °C for 3 h, the solid was washed, dried at 80 °C and calcined at 550 °C for 4 h.

#### 2.2.4. Suspension (Su)

Cordierite pellets were immersed in an aqueous slurry of 20 wt% of Ce_0.2_Zr_0.8_O_2_ powder, 20 vol% molten (70 °C) Brij 56 (Sigma Aldrich, Steinheim, Germany) and 2.8 M HNO_3_, dried and air blown. This coating and drying process was performed five times before calcination at 550 °C for 4 h.

#### 2.2.5. Double Deposition: A Combination of Wet Impregnation and Suspension (DD)

Cordierite pellets were immersed in an aqueous solution of 0.3 M Ce(NO_3_)_3_ and 1.2 M ZrOCl_2_ and dried at 120 °C for 15 min. This wet impregnation was repeated 10 times, after which the coated cordierite was calcined at 300 °C for 2 h. Subsequently the pellets were immersed in an aqueous slurry of 20 wt% of Ce_0.2_Zr_0.8_O_2_ powder, 20 vol% molten (70 °C) Brij 56 (Sigma Aldrich) and 2.8 M HNO_3_, dried and air blown. This coating and drying process was performed five times before calcination at 550 °C for 4 h.

### 2.3. Preparation of the Complete Catalyst

The active phase, including MnO_2_–NiO–Co_3_O_4_ mixed oxides (mole ratio 2:3:3) was deposited on the Ce_0.2_Zr_0.8_O_2_/cordierite samples by wet impregnation.

Suitable amounts of Mn(NO_3_)_2_ solution (58 wt%, Merck), Ni(NO_3_)_2_·6H_2_O (99.0 wt%, Merck), and Co(NO_3_)_2_·6H_2_O (99.0 wt%, Merck) were dissolved in distilled water. The total concentration of all solutes in the final solution was 747 g/L. After drying at 120 °C for 30 min, the warm as prepared Ce_0.2_Zr_0.8_O_2_/cordierite samples, prepared by the double deposition method (DD), were immersed in the above solution for 5 min. The wet pellets were dried until completely dry. This procedure was repeated five times.

Finally, the impregnated samples were heated at a heating rate of 3 °C/min till 550 °C for 3 h.

For comparison, the same active phase was also impregnated on bare cordierite pellets (CordxH8) by the same procedure. The catalysts impregnated on bare cordierite and on Ce_0.2_Zr_0.8_O_2_/cordierite were symbolized as Ca.2 and Ca.3, respectively. The amount of MnO_2_–NiO–Co_3_O_4_ mixed oxides on cordierite and on Ce_0.2_Zr_0.8_O_2_/cordierite after impregnation was approximately 6 wt%. The amount of Ce_0.2_Zr_0.8_O_2_ on cordierite, and MnO_2_–NiO–Co_3_O_4_ on support/substrate, were determined by weighting the catalysts before and after the loading. The wt% loading was calculated as the ratio of the difference between the weight of the catalyst after and before the loading to the weight of the catalyst before the loading.

### 2.4. Material Characterization

The morphology of bare cordierite, Ce_0.2_Zr_0.8_O_2_-coated cordierite and the final catalyst was investigated by SEM (Scanning Electron Microscope) on a FEI-QUANTA 200 microscope (FEI, Hillsboro, OR, USA). The identification of the crystalline phases was carried out by XRD (X-ray Diffraction) on a D8 Advance Bruker device (Bruker, Karlsruhe, Germany), CuKα radiation, step scan = 0.03. The specific surface areas of the products were measured with a Micromeritics Gemini T88 (Micromeritics, Norcross, GA, USA). X-ray photoelectron spectroscopy (XPS) measurements were carried out using a S-Probe monochromatized XPS spectrometer from Surface Science Instruments (VG) with an Al Kα X-ray (1486.6 eV) monochromatic source. The measured surface was 250 μm by 1000 μm with a flood gun set to 3 eV. Experimental data were processed using the software package CasaXPS (Casa Software Ltd., Teignmouth, UK). All spectra were calibrated for a carbon 1 s peak at 284.6 eV.

Catalytic activities were measured in a micro–reactor set up with an internal diameter of 0.4 cm and a length of 60 cm. Unsupported MnO_2_–NiO–Co_3_O_4_ active phase was used as powder (particle sizes were 250–300 μm). Supported catalysts on cordierite were used as pellet (5 mm in diameter, 3 mm in thickness). The weight of active phase was 0.02 g catalyst in all case. The total gas flow was 0.0542 mmol/s at a pressure of 1 atm. The volume composition of the gas flow was 2.6% CO, 7.7% O_2_, 1.5% C_3_H_6_, 1.9% NO and the reaction temperatures ranged from 150 °C to 550 °C. The experiment data were obtained when the reaction reached to a stable condition and the experiments were performed in an oxidation environment (with the presence of O_2_ and NO) in order to prevent the influence of the reduction of NiO to Ni on the activity. Analysis of the propylene, oxygen, nitrogen oxide and carbon dioxide was performed using an on-line Focus–Thermo Scientific gas chromatograph with a thermal conductivity detector (TCD). NO*_x_* and C_3_H_6_ were detected with a column of 80/100 chromosorb and a column of carbowax 20 M in series while CO_2_, CO, O_2_ were detected with a column of 60/80 carboxen and a column of 80/100 porapak in series.

## 3. Results and Discussion

### 3.1. Influence of Deposition Methods on Surface Areas of Ce_0.2_Zr_0.8_O_2_/Cordierite Samples

In Table 1, the surface areas of Ce_0.2_Zr_0.8_O_2_/cordierite samples are listed, together with Ce_0.2_Zr_0.8_O_2_ powder obtained by treating as prepared solutions or slurries (without immersed cordierite pellets) under the same preparation procedures for comparison.

For the *in situ* solid combustion method, the highest loading is obtained when the sample is treated at 420 °C*.* This did however not result in a high surface area, which is in an agreement with the literature [8]. While pure correlated powder exhibits surface areas of 46 and 50 m^2^/g, deposition on cordierite results in a reduction of the surface area from 20 m^2^/g for pure cordierite till 14 m^2^/g (420 °C) or 18 m^2^/g (120 °C) for the Ce_0.2_Zr_0.8_O_2_-coated pellets. This indicates that the higher the heating temperature, the closer the Ce_0.2_Zr_0.8_O_2_ grains will be packed on the surface of the cordierite.

With the aim to obtain high loading contents of the ceria—zirconia layer on the cordierite substrate, hybrid deposition was applied. It was expected that a sol of Ce(NO_3_)_3_ and ZrOCl_2_ could act as a binder for improved attachment of high surface area-Ce_0.2_Zr_0.8_O_2_ powder on the cordierite surface. However, Ce_0.2_Zr_0.8_O_2_ loading contents of the sample prepared by the hybrid deposition method was low compared to other methods (only 0.98%). Ce_0.2_Zr_0.8_O_2_ powder obtained under the same preparation procedure, and, thus, heat-treated at 550 °C, also possesses a much lower surface area (33 m^2^/g) compared to uncalcined as-prepared Ce_0.2_Zr_0.8_O_2_ powder (200 m^2^/g). As there is little material deposited on the surface of the pellet, the surface area only diminishes slightly (18 m^2^/g).

**Table 1:** Surface area of Ce_0.2_Zr_0.8_O_2_/cordierite samples prepared by different deposition method.

Samples	Deposition method	BET surface area (m^2^/g)		wt.% loading of Ce_0.2_Zr_0.8_O_2_ on cordierite
		*Coated pellets*	*Ce_0.2_Zr_0.8_O_2_ powder*	
*CordxH8*	No deposition	20	-	-
*SC-120*	*In situ* solid combustion, oven temperature 120 °C	18	50	3.84
*SC-420*	*In situ* solid combustion, oven temperature 420 °C	14	46	10.09
*HD*	Hybrid deposition	18	33	0.98
*Seed*	Secondary growth on Ce_0.2_Zr_0.8_O_2_ seeds	8	46 (seeding) 117 (hydrothermal)	-
*Su*	Suspension	24	74	3.5
*DD*	Double deposition method	25	56	4.39

Ce_0.2_Zr_0.8_O_2_ powder prepared by a hydrothermal method usually exhibits high surface area. However, due to the compact surface of cordierite and the differences in the crystal structure between the cordierite and the ceria—zirconia mixed oxide, it is very difficult for the Ce_0.2_Zr_0.8_O_2_ to grow on the cordierite surface. To improve it, the method which was called secondary growth on Ce_0.2_Zr_0.8_O_2_ seeds was performed. This method aimed to form a layer of Ce_0.2_Zr_0.8_O_2_ seeds on the cordierite surface by an *in situ* solid combustion method prior to a hydrothermal method. Ce_0.2_Zr_0.8_O_2_ seeds were expected to act as nuclei for the growth of a hydrothermally synthesized layer of high-surface-area-Ce_0.2_Zr_0.8_O_2_. However, the results show that the BET surface area of the sample obtained from secondary growth (8 m^2^/g) is even lower than that of the sample prepared by the solid combustion method (14 m^2^/g). The first grown Ce_0.2_Zr_0.8_O_2_ layer was formed using the *in situ* solid combustion method, which has an advantage of high loading content, but a disadvantage of loose adhesion of the Ce_0.2_Zr_0.8_O_2_ layer on the cordierite surface. Thus, during the second growth by hydrothermal synthesis, the cluster of Ce_0.2_Zr_0.8_O_2_ was detached from the surface due to the presence of the hydrothermal solution. It seems that no Ce_0.2_Zr_0.8_O_2_ particles, formed during the hydrothermal step, could be deposited on the seed layer.

The viscosity of the slurry plays an important role to the efficiency to attach solid particles from a slurry on the surface of a substrate. Therefore Brij-56 was used as a binder. Although the total amount of Ce_0.2_Zr_0.8_O_2_ attached to the substrate in this suspension method (3.5 wt%) is lower than what was reached using the *in situ* combustion method at 420 °C, the final surface area was higher (24 m^2^/g). However, many cracks are observed on the surface of the Ce_0.2_Zr_0.8_O_2_ layer.

With the aim to avoid crack formation in the Ce_0.2_Zr_0.8_O_2_ layer deposited by suspension in a slurry, a double deposition method was investigated. First a smooth layer of Ce_0.2_Zr_0.8_O_2_ is formed by immersion in a Ce(NO_3_)_3_ and ZrOCl_2_ containing solution, subsequently the above described suspension method (d) is applied. The BET surface area of this sample is comparable (25 m^2^/g) to that of the sample prepared by the suspension method since both methods use the same Ce_0.2_Zr_0.8_O_2_ slurry and the synthesis procedures were not significantly different. However, less cracks have been observed on the surface of the sample prepared by this double deposition method, showing that better conditions for the adhesion of the rough Ce_0.2_Zr_0.8_O_2_ layer were created. The double deposition method also has the advantage of a slightly higher loading content of Ce_0.2_Zr_0.8_O_2_ compared to that obtained by the suspension method (4.39 wt%).

### 3.2. Influence of the Deposition Methods on the Morphology of Ce_0.2_Zr_0.8_O_2_/Cordierite Samples

**Figure 2:**
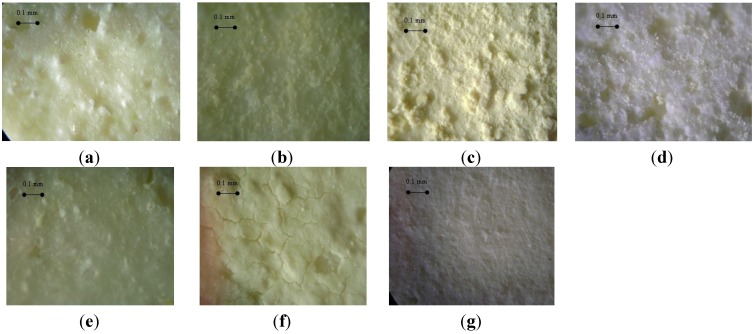
Microscopic images of Ce_0.2_Zr_0.8_O_2_/cordierite samples prepared by different impregnation methods (magnification 40 times): (**a**) bare cordierite substrate (CordxH8); (**b**) solid combustion at 120 °C (SC-120); (**c**) solid combustion at 420 °C (SC-420); (**d**) hybrid deposition (HD); (**e**) Secondary growth on Ce_0.2_Zr_0.8_O_2_ seeds (Seed); (**f**) suspension (Su); (**g**) double deposition (DD).

Surfaces of Ce_0.2_Zr_0.8_O_2_/cordierite samples prepared by different methods observed under a microscope are presented in Figure 2. It can be seen that the surface of the samples prepared by solid combustion at 120 °C (SC-120), hybrid deposition (HD) and the seed method are not very different from that of the unimpregnated cordierite (CordxH8). This confirms that these methods did result in an appreciable amount of Ce_0.2_Zr_0.8_O_2_ on the surface of the cordierite. An inhomogeneous surface was seen on the sample prepared by solid combustion at 420 °C. The adhesion between Ce_0.2_Zr_0.8_O_2_ layers and the cordierite substrate showed to be extremely loose in this sample, presumably because the evaporation of water during the deposition process at high temperature was too rapid. It was also agreed in the literature that crumbling Ce_0.2_Zr_0.8_O_2_ layer can be detached easily from the cordierite using *in situ* combustion method [8].

In the sample prepared by the suspension method many cracks were observed. Although the use of a binder in the suspension method was able to produce a more viscous slurry resulting in a higher Ce_0.2_Zr_0.8_O_2_ loading content compared to some of the other methods, burning of the polymer binder during the calcination step may be the reason for the presence of these cracks. On the other hand, the double deposition method resulted in the smoothest surface. The presence of a previously formed Ce_0.2_Zr_0.8_O_2_ layer prior to the deposition of the Ce_0.2_Zr_0.8_O_2_ layer from a slurry may prevent the growth of cracks on the surface of the final samples. Agrafiotis *et al.* also deposited γ-Al_2_O_3_ on cordierite by a similar suspension synthesis [9] and found that small particle size γ-Al_2_O_3_ lead to a homogeneous washcoat layer with good adhesion properties and a surface area of about 50 m^2^/g. However, the use of Ce_0.2_Zr_0.8_O_2_ instead of γ-Al_2_O_3_ in this work faced to a less linked Ce_0.2_Zr_0.8_O_2_ particles. To solve that problem, Brij56 binder was added as a linkage agent but also lead to a less homogeneous surface with crack. Therefore, double deposition method, which has not been tested previously in the literature, was the used to prevent successfully the crack and to obtain as homogeneous with good adhesion washcoat layer, as obtained in the literature for γ-Al_2_O_3_.

**Figure 3:**
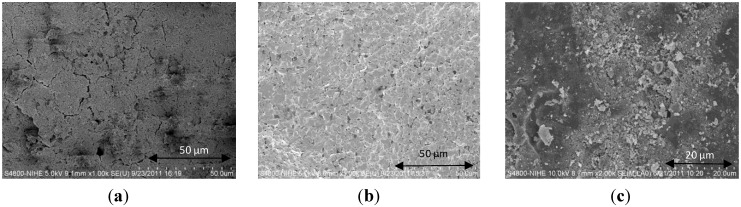
SEM images of Ce_0.2_Zr_0.8_O_2_/cordierite samples prepared by (**a**) suspension method-Su; (**b**) double deposition method—DD and (**c**) acid treated cordierite—CordxH8.

Morphologies of the samples prepared by the suspension and the double deposition method were also analyzed by SEM (Figure 3a,b). The change in morphology compared to unimpregnated cordierite (Figure 3c), is clearly seen. The Ce_0.2_Zr_0.8_O_2_ layer prepared by the suspension method possessed much finer particles than the one prepared by the double deposition method. This may be due to the fact that the double deposition method includes two calcination steps of two continuous Ce_0.2_Zr_0.8_O_2_ layers, thus, increasing sintering of particles.

### 3.3. Characterization of the Complete Catalyst

**Figure 4:**
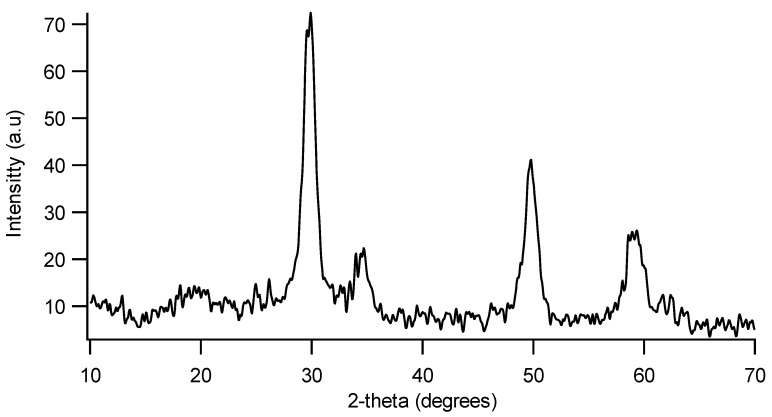
XRD pattern of Ce_0.2_Zr_0.8_O_2_ powder obtained by the same synthesis procedure as that deposited on cordierite substrate.

Since the impregnation of Ce_0.2_Zr_0.8_O_2_ on cordierite substrates using a double deposition method resulted in the most homogeneous and stable layer, the samples prepared by this method were selected for further deposition of the catalytic active phase. Since the content of the Ce_0.2_Zr_0.8_O_2_ layer on cordierite substrate was low (4.39 wt%), the formation of Ce_0.2_Zr_0.8_O_2_ on cordierite substrate was proved by XRD characterization of the precursor powder previous synthesized by hydrothermal method, which was calcined under the same procedure as the Ce_0.2_Zr_0.8_O_2_/cordierite sample. The characterization showed that Ce_0.2_Zr_0.8_O_2_ was successfully formed (Figure 4). The powder possesses the same crystal structure like CeO_2_ but with the two theta degrees shifted to the higher value, showing the replacement of Zr atoms for Ce, which is in an agreement with the literature [21].

**Figure 5:**
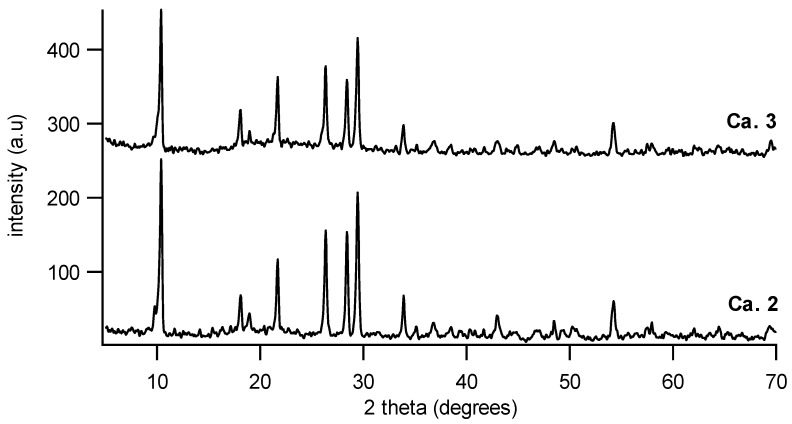
XRD pattern of MnO_2_–NiO–Co_3_O_4_/cordierite (Ca.2) and MnO_2_–NiO–Co_3_O_4_/Ce_0.2_Zr_0.8_O_2_/cordierite (Ca.3).

The XRD patterns of the MnO_2_–NiO–Co_3_O_4_ active phase on cordierite sample (Ca.2) and on the Ce_0.2_Zr_0.8_O_2_/cordierite sample (Ca.3) were measured. Due to low loading content of Ce_0.2_Zr_0.8_O_2_ (4 wt%) and MnO_2_–NiO–Co_3_O_4_ (6 wt%), the main peaks are those of pure cordierite (Figure 5). Thus, there is almost no difference in XRD patterns of the sample with (Ca.3) and without Ce_0.2_Zr_0.8_O_2_ layer (Ca.2).

**Figure 6:**
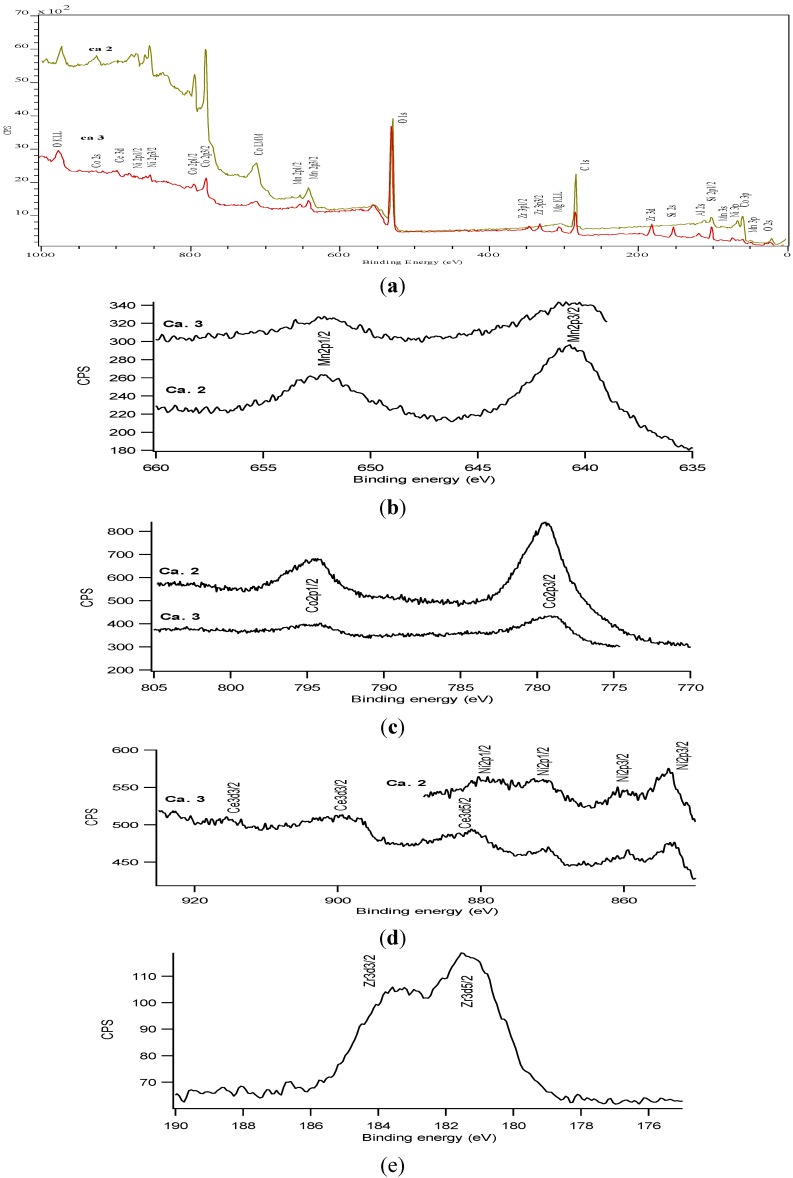
XPS spectra of the as-prepared sample Ca.2 (MnO_2_–NiO–Co_3_O_4_/cordierite) and Ca.3 (MnO_2_–NiO–Co_3_O_4_/Ce_0.2_Zr_0.8_O_2_/cordierite): (**a**) survey spectra; (**b**) Mn region; (**c**) Co region; (**d**) Ni–Ce region; (**e**) Zr region.

XPS results in Table 2 and Figure 6 show the presence of components of the catalyst (Mn, Ni, Co), the components of the supporting Ce_0.2_Zr_0.8_O_2_ layer (Ca.3) and the cordierite substrate (Si, Al). Based on binding energies and shape of selected peaks and after comparison with peaks from a mechanical mixture of MnO_2_, Co_3_O_4_ and NiO we can conclude that Co is present as Co_3_O_4_ (Co 2p3/2 at 779.8 eV) and oxidation states of other elements are Ni^2+^ (Ni 2p3/2 at 854 eV and its satellite at 860 eV), Mn^4+^ (Mn2p3/2 at 641 eV). Characteristic peaks of the elements of the supporting layer show that Ce^4+^ (presence of characteristic peak at 916 eV), and Zr^+4^ (Zr 3d5/2 at 181.2 eV and Zr 3d3/2 at 183.6 eV) are present [22].

**Table 2:** Atomic compositions (%) of components in Ca.2 and Ca.3 catalysts by XPS.

Elements	Ca.2 MnO_2_–NiO–Co_3_O_4_/cordierite	Ca.3 MnO_2_–NiO–Co_3_O_4_/Ce_0.2_Zr_0.8_O_2_/cordierite
*Mn*	14.6	21.4
*Co*	42.5	29.8
*Ni*	42.9	22.0
*Ce*	0	8
*Zr*	0	18.8

**Figure 7:**
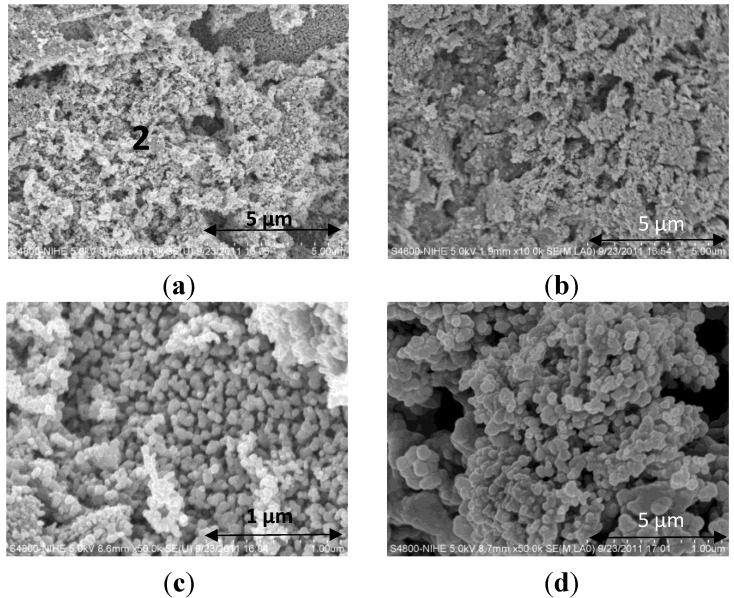
SEM images of final catalysts: Ca.2 (MnO_2_–NiO–Co_3_O_4_/cordierite) and Ca.3 MnO_2_–NiO–Co_3_O_4_/Ce_0.2_Zr_0.8_O_2_/cordierite: (**a**) Ca.3 at magnification 10000×; (**b**) Ca.2 at magnification 1000×; (**c**) Ca.3, region 2 at magnification 50000×; (**d**) Ca.2 at magnification 50000×.

SEM images of the final catalysts are presented in Figure 7. In the catalyst with a Ce_0.2_Zr_0.8_O_2_ support layer two different parts are observed, where part 1, seems to lay underneath and incompletely covered by Part 2 (Figure 7a). EDX (Energy-dispersive X-ray spectroscopy) point analysis confirms that Part 1 can be assigned to the Ce_0.2_Zr_0.8_O_2_ support layer, while part 2 is the MnO_2_–NiO–Co_3_O_4_ active layer. At higher magnification (Figure 5c,d), the two catalysts showed morphology with spherical particles of about 100 nm, which proves that the MnO_2_–NiO–Co_3_O_4_ active layer retains its specific morphology after deposition on the cordierite substrate (Ca.2), as well as on the Ce_0.2_Zr_0.8_O_2_ support layer (Ca.3).

### 3.4. Catalytic Activities of the Final Catalysts

**Figure 8:**
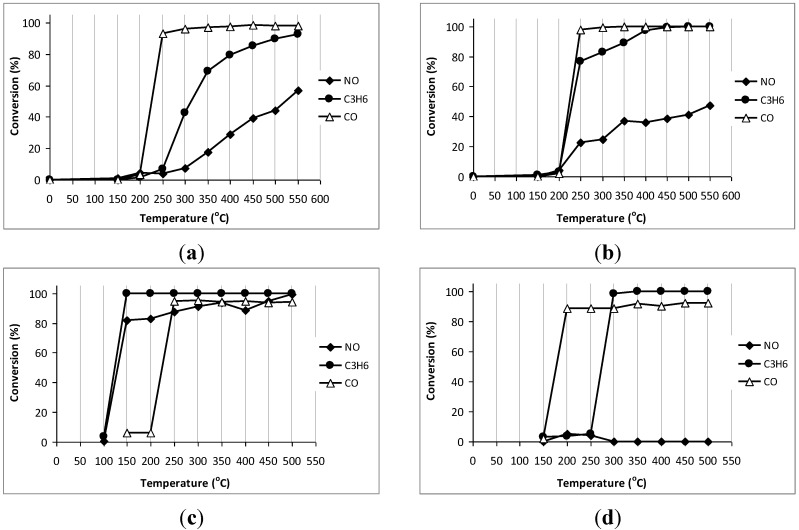
Catalytic activities for the treatment of CO, C_3_H_6_, NO of different catalysts: (**a**) MnO_2_–NiO–Co_3_O_4_/cordierite (Ca.2); (**b**) MnO_2_–NiO–Co_3_O_4_/Ce_0.2_Zr_0.8_O_2_/cordierite (Ca.3); (**c**) unsupported MnO_2_–NiO–Co_3_O_4_ active phase; (**d**) commercial noble catalyst on cordierite substrate (CATCO-920).

The ability to treat toxic components in exhaust gases (NO, CO, C_3_H_6_) of the MnO_2_–NiO–Co_3_O_4_/cordierite and MnO_2_–NiO–Co_3_O_4_/Ce_0.2_Zr_0.8_O_2_/cordierite catalysts are demonstrated in Figure 8a,b. It was shown that bare cordierite and the Ce_0.2_Zr_0.8_O_2_ on cordierite exhibit almost no activity to treat NO, CO and C_3_H_6_. Meanwhile, powder unsupported MnO_2_–NiO–Co_3_O_4_ exhibited excellent activity: 90%–100% conversion of all CO, NO and C_3_H_6_ from 250 °C (Figure 8c). When MnO_2_–NiO–Co_3_O_4_ was deposited on cordierite, the catalytic activity increases significantly compared to the substrate and support/substrate while slightly decreased compared to the powder unsupported catalytic active phase. The Ca.2 catalyst was able to convert 100% CO at low temperature (250 °C), 80%–100% C_3_H_6_ at temperatures from 400 °C onwards and 40% NO from 450 °C onwards. The fact that CO conversion on Ca.2 was about 5% higher than that on unsupported active phase MnO_2_–NiO–Co_3_O_4_ did not indicate a higher activity for CO oxidation because this different value may fall in the range of experimental error. On the other hand, when a slightly decrease of CO conversion occurs together with an increase of C_3_H_6_ conversion, it is the evidence that the oxidation of C_3_H_6_ seems to be the priority than CO on the unsupported catalysts, which suggests that the unsupported catalyst was more advanced (due to possess more catalytic active sites) since C_3_H_6_ is known to be more difficult to be oxidized than CO. Due to the presence of less active sites in supported catalysts (Ca.2, Ca.3), NO reduction and C_3_H_6_ oxidation also became more difficult than those in the unsupported one. The Ca.3 sample exhibited higher activities than that on Ca.2, a conversion of 40% NO was reached at low temperature (350 °C), 80% conversion of C_3_H_6_ was already obtained at 250 °C and, at 400 °C, almost 100% C_3_H_6_ had been converted. Thus, the presence of the Ce_0.2_Zr_0.8_O_2_ support, indeed, improved the catalytic activity of the catalyst. The higher activity of the catalyst with Ce_0.2_Zr_0.8_O_2_ support may be assigned for the high oxygen storage capacity of Ce_0.2_Zr_0.8_O_2_ as known from literature [23,24,25], which may help to provide rapidly the oxygen used for the oxidation reaction. It was also known from our experiment that the use of Ce_0.2_Zr_0.8_O_2_ support resulted in a better catalytic activity than the use of γ-Al_2_O_3_ support, which was not reported as a material with high oxygen storage capacity. Here, the role of the surface area may not be significant since both catalysts exhibit a rather equal surface area (surface area of Ca.2 and Ca.3 catalysts is 29 m^2^/g and 23 m^2^/g, respectively).

Compared to a commercial catalyst for a gasoline engine, prepared from noble metals on cordierite substrates (content of cordierite in the substrate: 100%, surface area: 33 m^2^/g, the support was based on Al-Ce-Zr mixed oxide, the active phase was based on Pt, Pd, Rh with the total loading content up to 2–3 wt%), the conversion of CO and C_3_H_6_ is comparable (Figure 8d). The lowest temperature for 100% C_3_H_6_ conversion on Ca.3 catalyst was 100 °C higher than that on the commercial catalyst. However, Ca.3 catalyst was able to convert 100% CO at a reasonably low temperature (250 °C) while the commercial catalyst was only able to convert about 90% CO. For NO conversion Ca.3 catalyst performed much better than the commercial catalyst (40% and 0%, respectively). The reason for the poor NO conversion of the commercial catalyst may be due to the fact that this commercial catalyst was produced to apply in gasoline engines, therefore the catalyst components were chosen mostly to treat hydrocarbons and CO while the treatment of NO was not cared for as it was different with diesel engines, the NO concentration in gasoline engine is normally low. Here, this commercial catalyst was still used for the comparison with our catalysts since our aim was producing catalysts for gasoline engines.

## 4. Conclusions

This paper investigated different methods to impregnate Ce_0.2_Zr_0.8_O_2_ supports on a cordierite substrate for application as three-ways catalysts. The double deposition method, a combination of wet impregnation and suspension in a Ce_0.2_Zr_0.8_O_2_-slurry resulted in the most homogeneous surface and a stable adhesion of the Ce_0.2_Zr_0.8_O_2_ layer on the substrate. The deposition of a MnO_2_–NiO–Co_3_O_4_ active phase on both bare cordierite and Ce_0.2_Zr_0.8_O_2_/cordierite resulted in a reasonably thick and uniform layer. It was shown that the catalytic activity of the sample with a Ce_0.2_Zr_0.8_O_2_ support layer was higher, which is allocated to its oxygen storage capacity. The activity of this catalyst for the treatment of CO and C_3_H_6_ was comparable to that of a commercial noble-metal-containing catalyst for a gasoline engine. Concerning NO conversion this MnO_2_–NiO–Co_3_O_4_ showed vastly better results.
